# Supplementary figures and images for: Accessibility of the unstructured α-tubulin C-terminal tail is controlled by microtubule lattice conformation
Source: eLife. 2026 Feb 9;14:RP109308. doi: 10.7554/eLife.109308 (PMC12885479; doi:10.7554/eLife.109308)

Figure 1E

Blot YL1/2 IgG

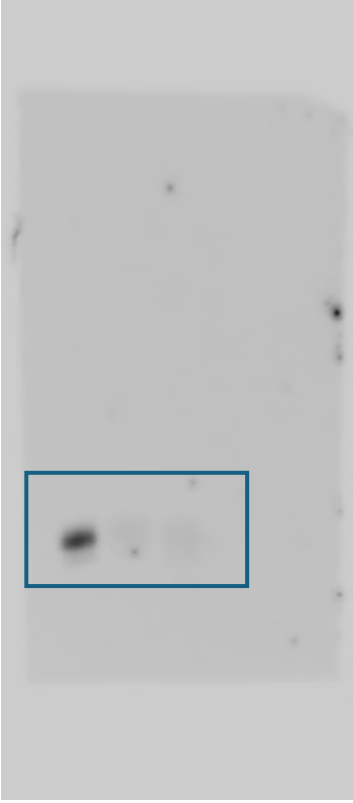

blot rMAb-YL1/2

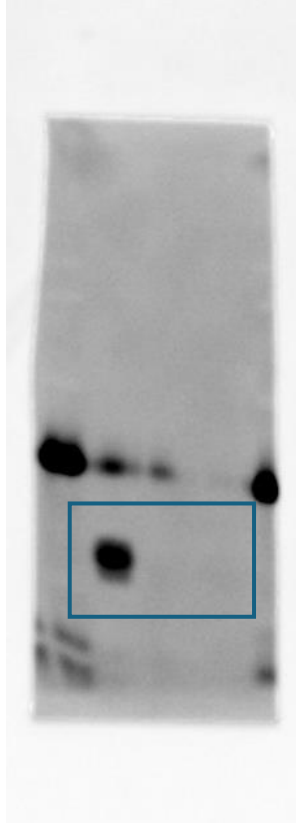

blot YL1/2<sup>Fab</sup>

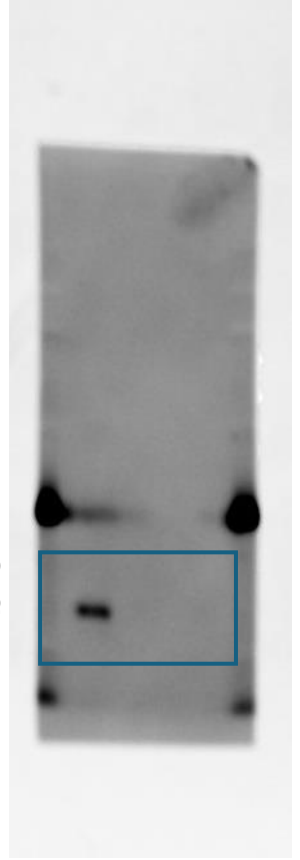

blot GST

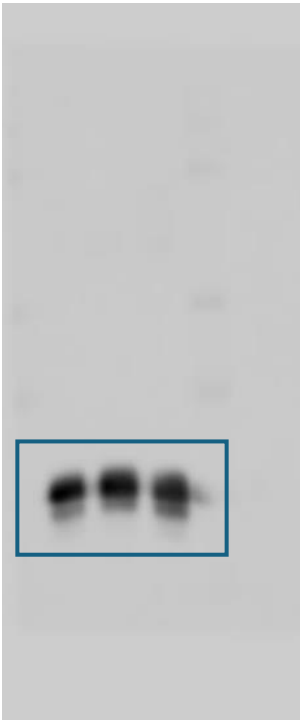

blot GST

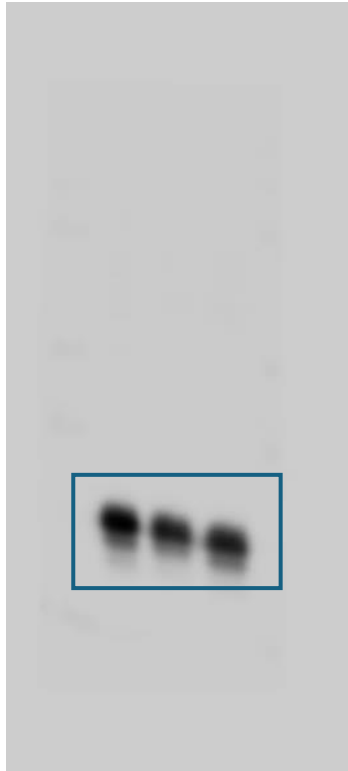

blot GST

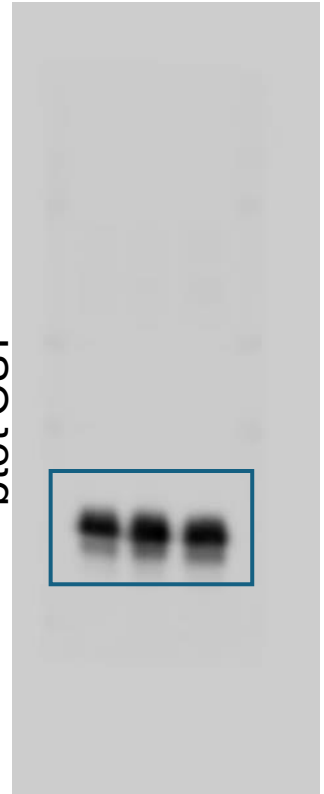

Supplement: Figure 1—source data 1. [file elife-109308-fig1-data1.zip › Figure1-sourcedata1-labelled/Figure1-sourcedata1-labelled.pdf]

Fig1-FigSupp1B

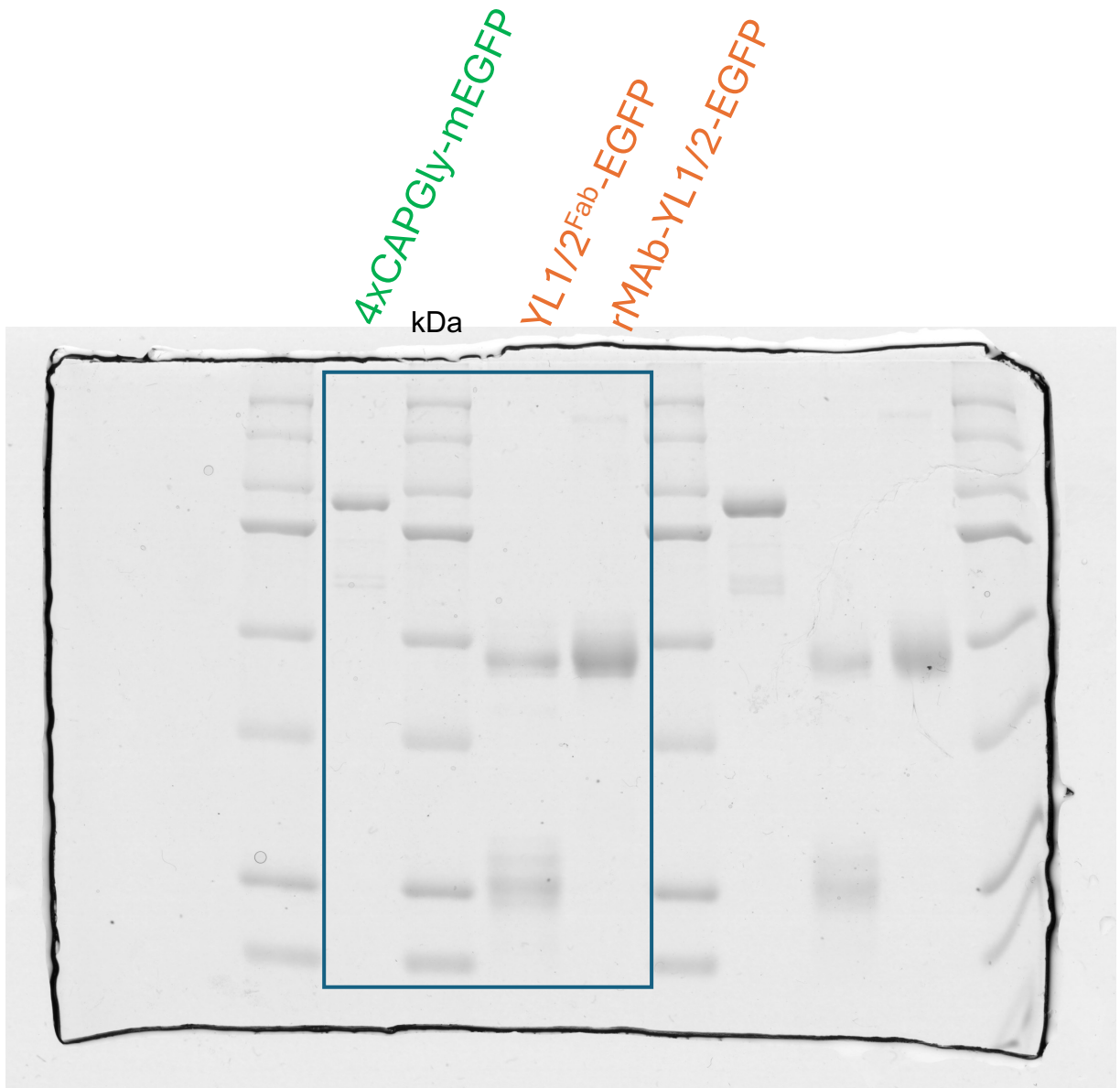

Fig1-Supp1C

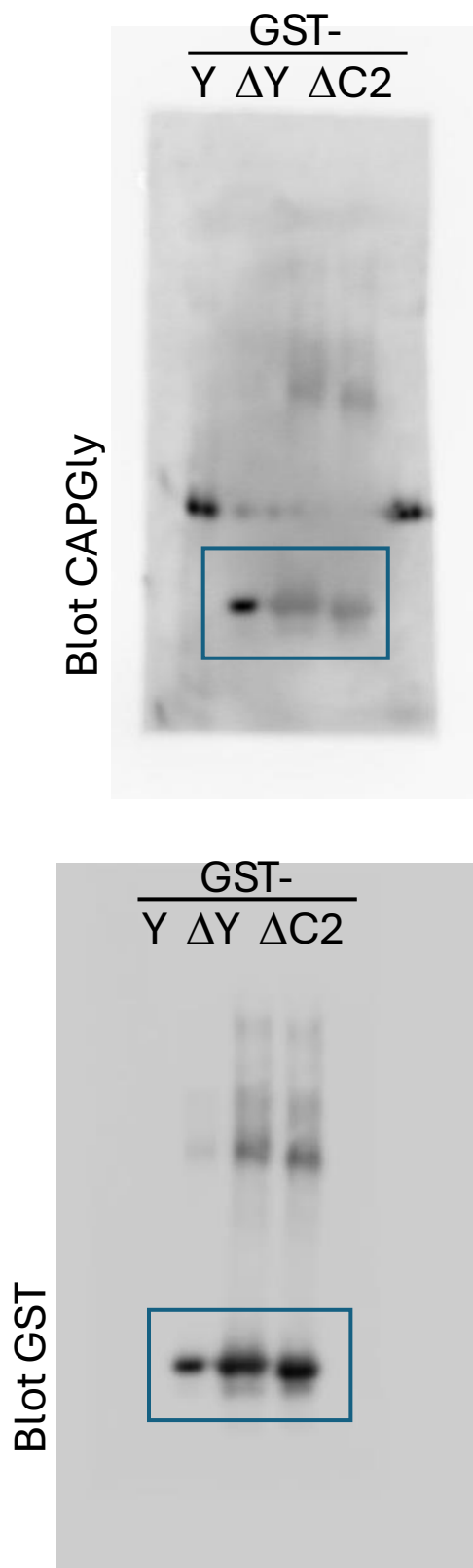

Supplement: Figure 1—figure supplement 1—source data 1. [file elife-109308-fig1-figsupp1-data1.zip › Figure1-FigSupp1-sourcedata1-labelled/Figure1-FigSupp1-sourcedata-labelled.pdf]

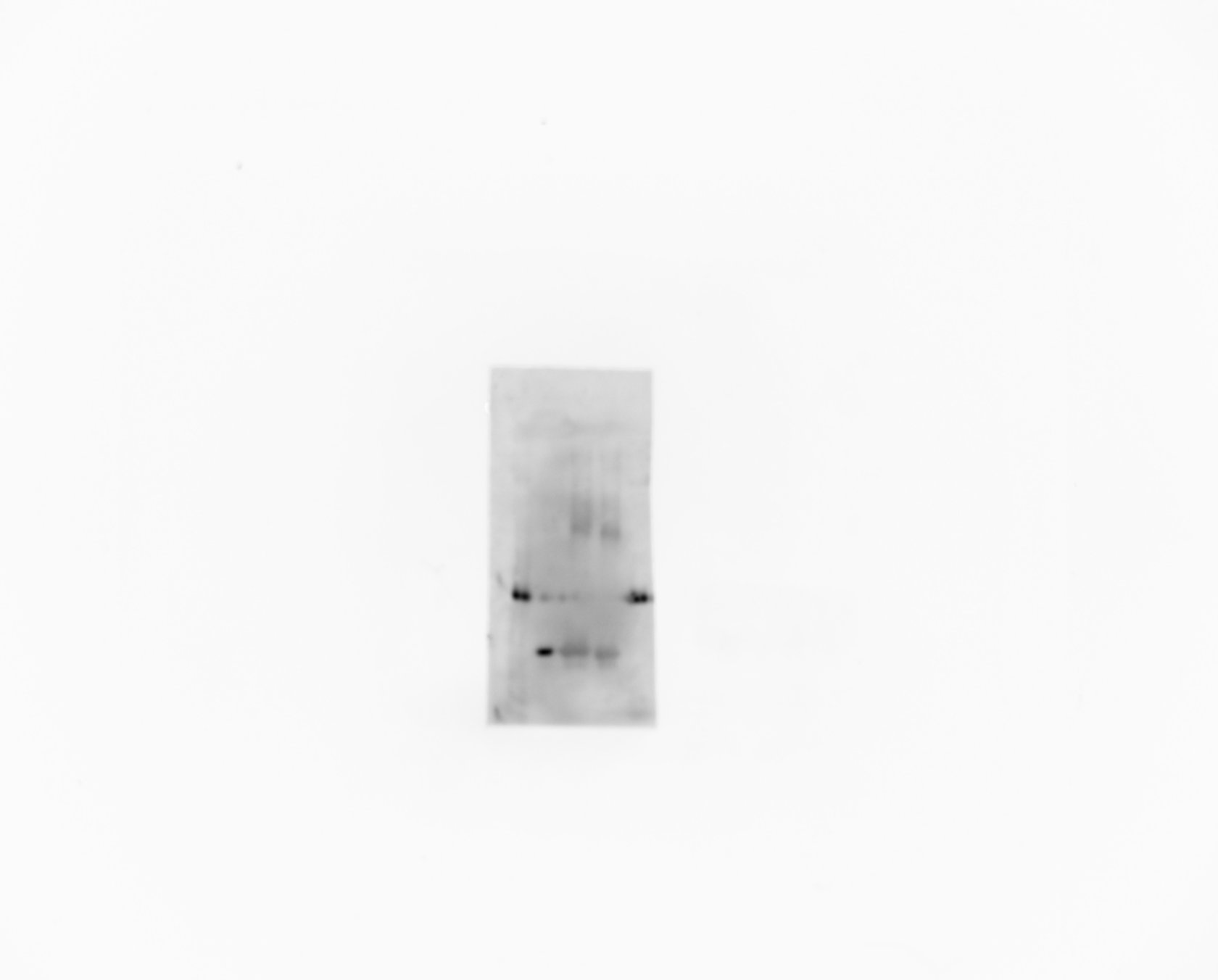

Supplement: Figure 1—figure supplement 1—source data 2. [file elife-109308-fig1-figsupp1-data2.zip › Figure1-FigSupp1-sourcedata1-original/Figure1-FigSupp1C_blot 4xCAPGly.tiff]

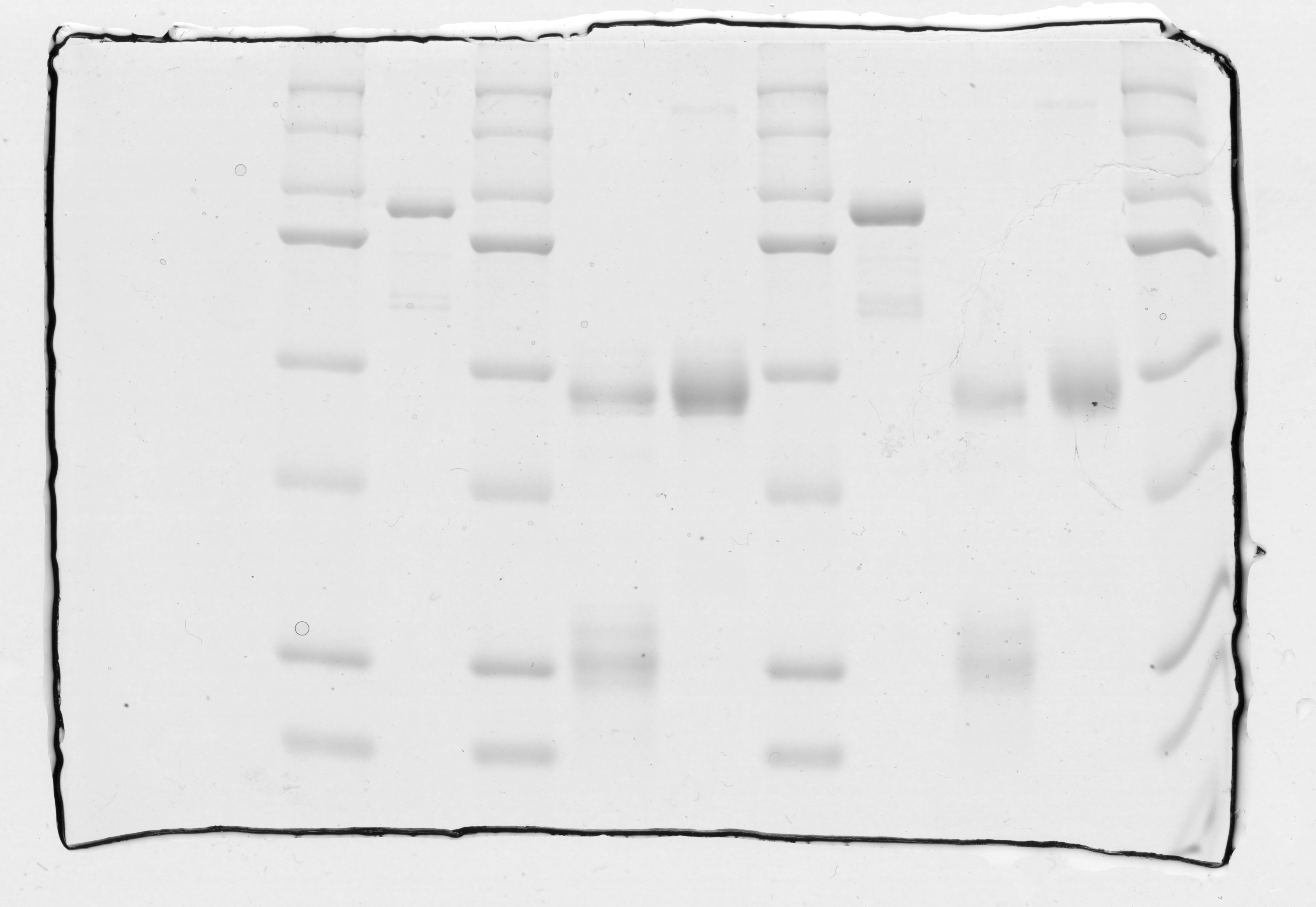

Supplement: Figure 1—figure supplement 1—source data 2. [file elife-109308-fig1-figsupp1-data2.zip › Figure1-FigSupp1-sourcedata1-original/Figure1-FigSupp1B_gel.tif]

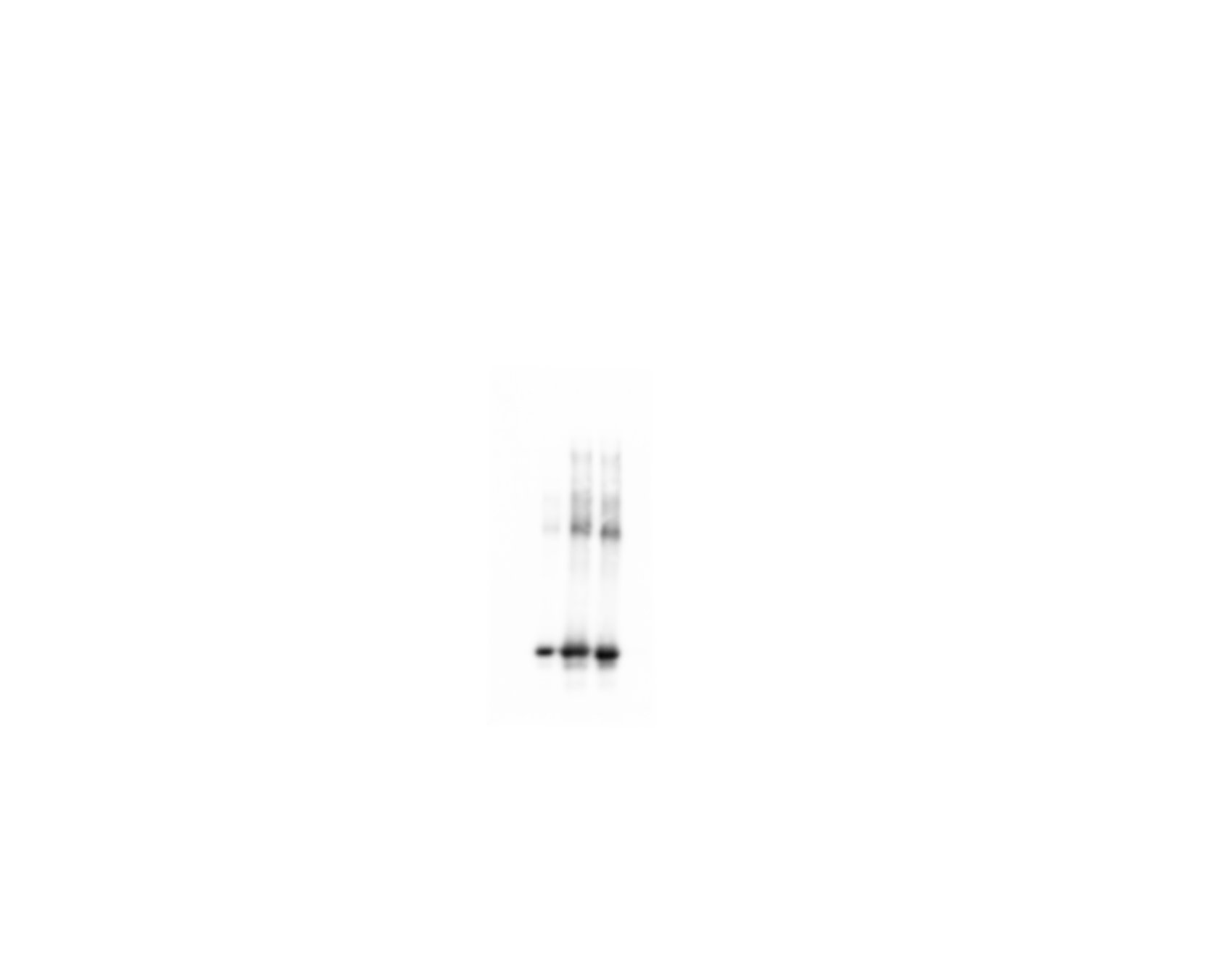

Supplement: Figure 1—figure supplement 1—source data 2. [file elife-109308-fig1-figsupp1-data2.zip › Figure1-FigSupp1-sourcedata1-original/Figure1-FigSupp1C_blot GST.tiff]
