# Supplementary material for: Accessibility of the unstructured α-tubulin C-terminal tail is controlled by microtubule lattice conformation
Source: eLife. 2026 Feb 9;14:RP109308. doi: 10.7554/eLife.109308 (PMC12885479; doi:10.7554/eLife.109308)

Blot PA-TubA1A

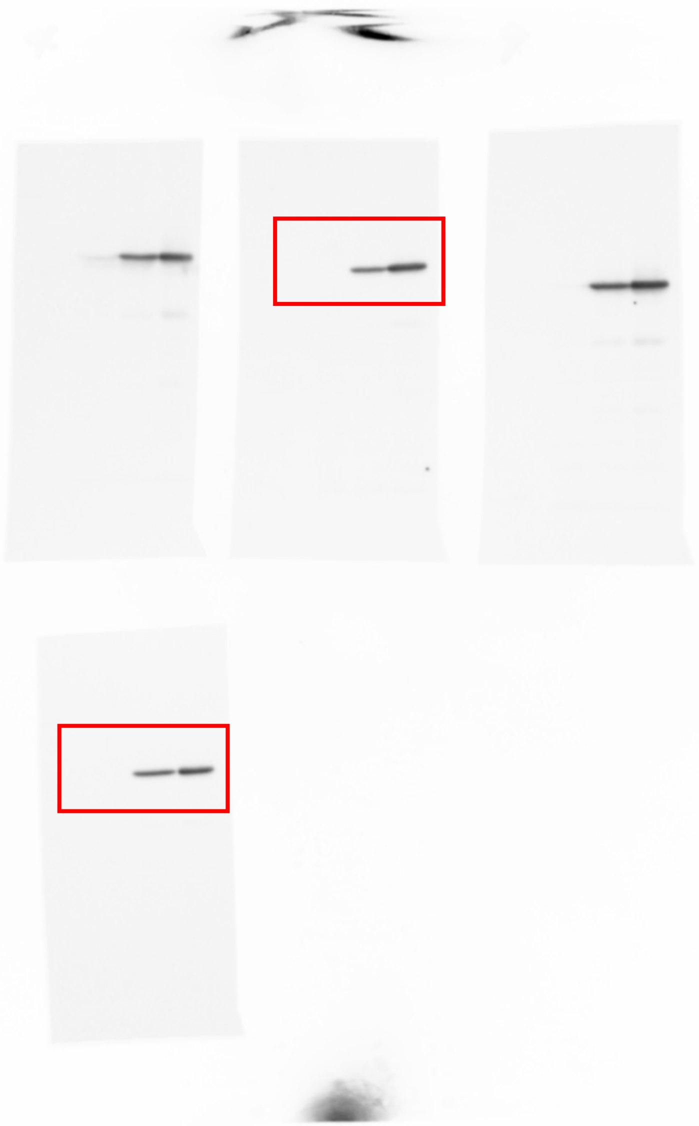

Blot total α-tubulin

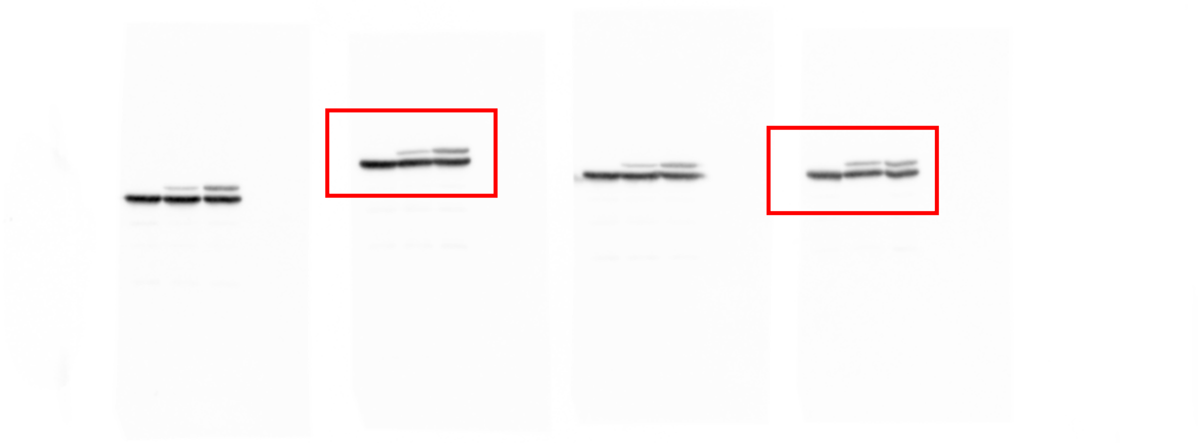

Blot Y-α-tubulin

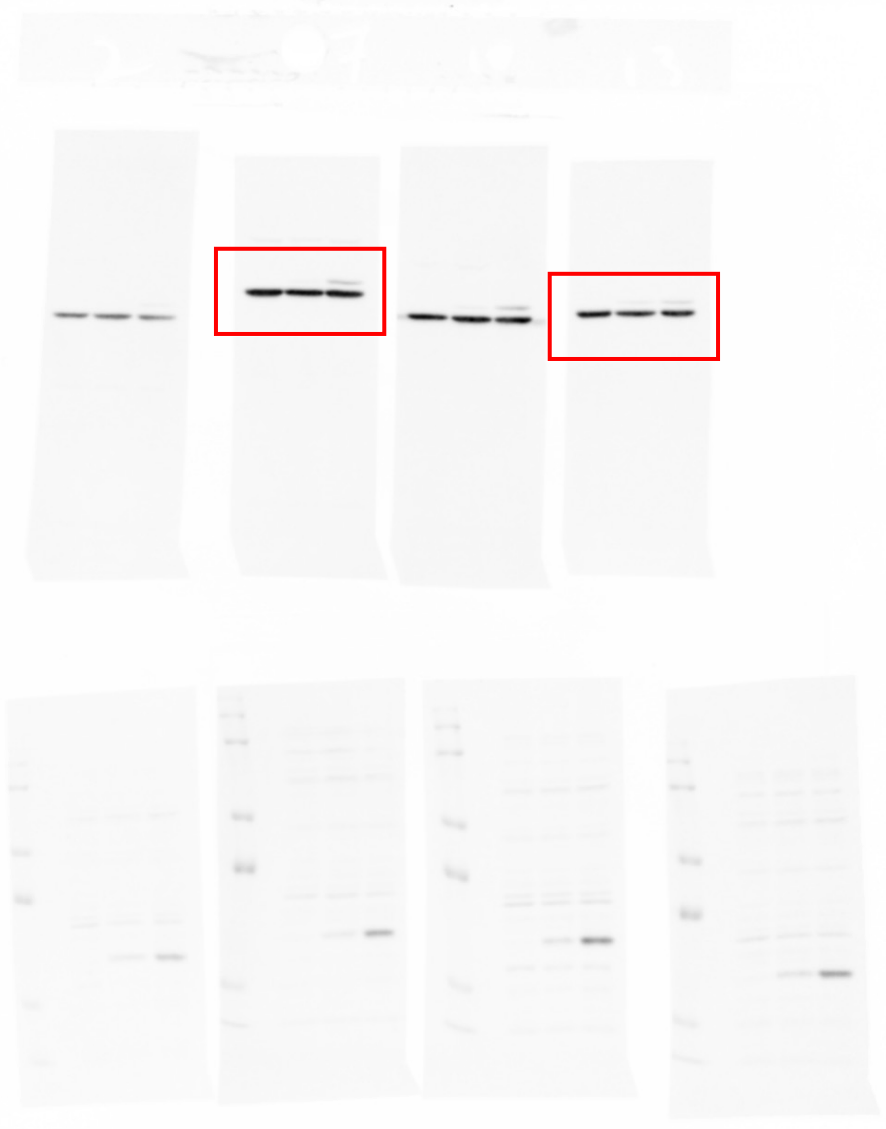

Blot Tag-RFP

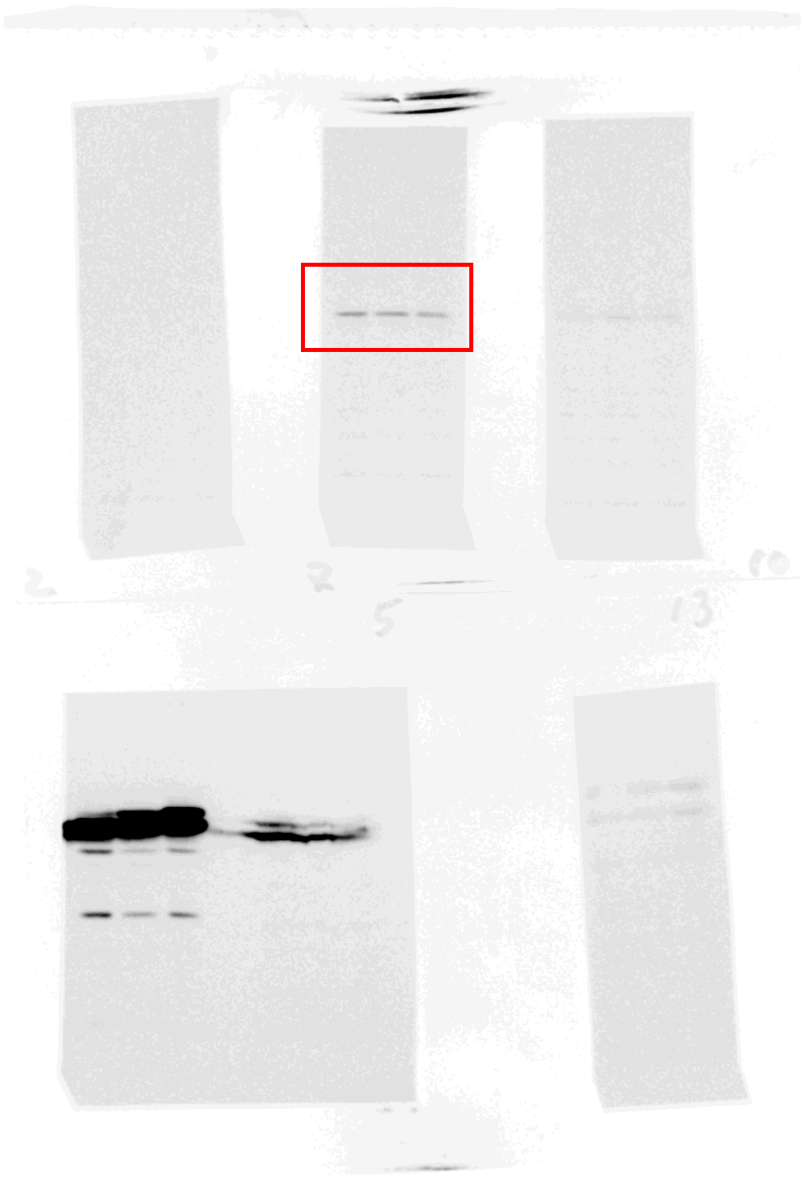

Blot mScarlet3

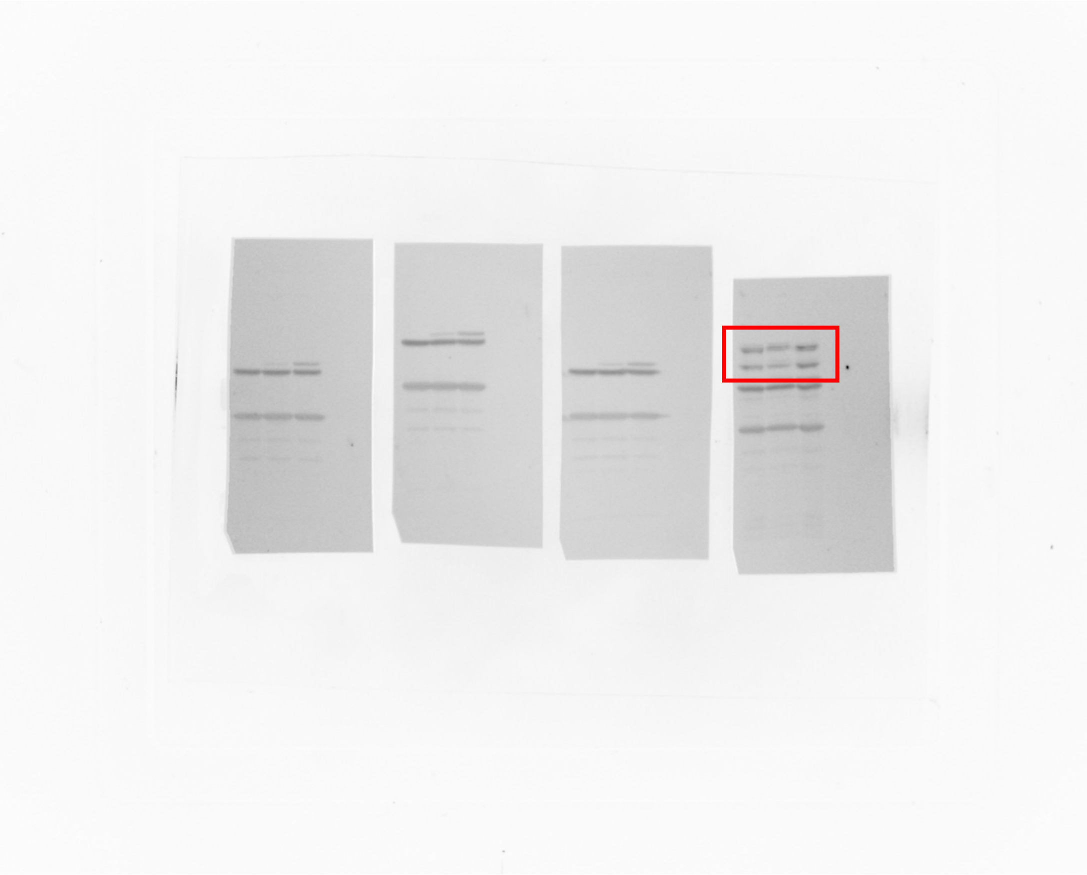

Blot GAPDH

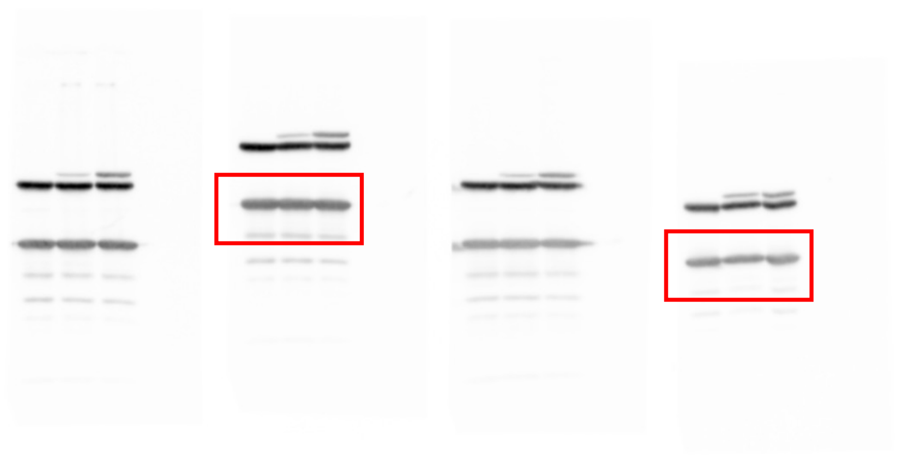

Supplement: Figure 5—figure supplement 1—source data 1. [file elife-109308-fig5-figsupp1-data1.zip › Figure5-FigSupp1-sourcedata1-labelled/Fig5-FigSupp1-sourcedata1-labelled.pdf]
